# Depression and schizophrenia

**DOI:** 10.4103/0019-5545.49458

**Published:** 2009

**Authors:** Chittaranjan Andrade

**Affiliations:** Department of Psychopharmacology, National Institute of Mental Health and Neurosciences, Bangalore - 560 029, India

## CME QUESTIONS

A) Schizophrenia and bipolar disorder are both associated with an increased risk of weight gain, incident diabetes mellitus, and the metabolic syndrome; at least a part of the risk is mediated by iatrogenic factors such as atypical antipsychotic medication and mood stabilizers. The relationship between depression and diabetes mellitus has also been studied. With this background, mark *True* or *False* against each of the following statements:

1. Depression is associated with an increased risk of incident diabetes mellitus.
2. Diabetes mellitus is associated with an increased risk of incident depression.
3. Depression is associated with superior treatment adherence to antidiabetic measures.
4. In diabetic samples, inactive persons are less likely to be depressed.
5. In diabetic samples, depressed persons are more likely to be inactive.
6. In diabetic samples, long-term glycemic control is no different between depressed and nondepressed patients.

B) Just as antidepressant augmentation strategies are employed in antidepressant-refractory depression, so too are antipsychotic augmentation strategies considered in antipsychotic-refractory schizophrenia. Lamotrigine is a broad-spectrum antiepileptic drug which has also demonstrated efficacy in the acute treatment of bipolar depression and in the maintenance therapy of rapid-cycling and nonrapid-cycling bipolar disorder. With this background, mark *True* or *False* against each of the following statements:

1. The rationale for the use of lamotrigine in schizophrenia is that the drug is a partial agonist at dopamine autoreceptors.
2. Lamotrigine augmentation of antipsychotic medication attenuates positive symptoms of schizophrenia.
3. Conventional doses of lamotrigine are associated with clinically significant increases in antipsychotic drug levels.
4. There is weak clinical evidence that lamotrigine augmentation may attenuate obsessive-compulsive disorder symptoms in patients with schizophrenia.

## CME ANSWERS

### A) Depression and diabetes mellitus

Answers: 1. True; 2. True; 3. False. 4. False; 5. True. 6. False.

Mezuk *et al.*[1] described a systematic review and meta-analysis of the relationship between depression and Type 2 diabetes. They identified 13 prospective studies of depression which excluded diabetes at baseline, and 7 prospective studies of diabetes which excluded depression at baseline. They found that depression was associated with a 60% increased risk of new-onset diabetes (Relative Risk [RR], 1.60; 95% confidence intervals [CI], 1.37-1.88) and diabetes was associated with a 15% increased risk of new-onset depression (RR, 1.15; 95% CI, 1.02-1.30).

Gonzalez *et al.*[2] described a systematic review and meta-analysis of the relationship between depression and treatment nonadherence in 47 studies of patients with type 1 and type 2 diabetes mellitus. They found that depression was significantly associated with treatment nonadherence; studies with better methodology demonstrated larger nonadherence effects.

Lysy *et al.*[3] described a systematic review of the relationship between physical activity and depressed mood in Type 2 diabetes patients. They identified 10 cross-sectional studies and 2 trials. They found that, in adults with Type 2 diabetes, inactive persons were 1.72-1.75 times more likely to be depressed, and depressed persons were 1.22-1.90 times more likely to be inactive.

Richardson *et al*.[4] examined the longitudinal effects of ICD-9 depression on glycemic control in 11,525 persons with Type 2 diabetes. The mean age of the sample was about 66 years. The sample was 97% male. The sample was followed up for a mean of 4.1 years. Depression was recorded in 6% of the sample. HbA1c values were found to be slightly (by a mean of 0.13%) but significantly higher in depressed persons at all time points during the study.

These findings suggest that, in depressed patients who are not diabetic, efforts should be made to modify lifestyle risk factors for diabetes in order to reduce the risk of future diabetes; and these patients should be monitored so that diabetes, if incident, is detected early. Likewise, diabetic patients should be screened for depression; and if they are not depressed, they should be monitored for incident depression. Finally, in depressed patients who are diabetic, efforts should be made to improve medication adherence and levels of physical activity so that short- and long-term glycemic control is improved.

### B) Lamotrigine augmentation in antipsychotic-refractory schizophrenia

Answers: 1. False; 2. False; 3. False; 4. True.

Lamotrigine has no action on dopamine receptors; however, as glutamatergic mechanisms have been proposed to explain schizophrenic symptoms[5] and as lamotrigine modulates glutamatergic neurotransmission, many studies have examined whether the addition of lamotrigine to an antipsychotic regime improves outcomes in antipsychotic-refractory schizophrenic patients.

In a double-blind, placebo-controlled, 14-week crossover trial, Tiihonen *et al*.[6] found that treatment-resistant schizophrenic inpatients (n=34) with clozapine-refractory psychotic symptoms showed small (6-7%) but statistically significant attenuation of positive (but not negative) symptom and general psychopathology ratings with lamotrigine (200 mg/day) augmentation of clozapine.

Kremer *et al*.[7] described a 10-week, randomized, double-blind, placebo-controlled study of lamotrigine (up to 400 mg/day) augmentation of conventional and atypical antipsychotic medication in 38 schizophrenic patients with treatment-resistant psychotic symptoms. An intent-to-treat analysis found that lamotrigine was no better than placebo. However, in the completer sample (n=31) lamotrigine significantly attenuated positive and general psychopathology ratings.

A Cochrane Review conducted in 2006[8] identified 5 trials (pooled n=537) of lamotrigine in schizophrenia. In these trials, lamotrigine was no better than placebo in response rates and in global response; however, positive symptoms, negative symptoms, and PANSS totals improved more with lamotrigine than with placebo. Cognitive measures differed little between lamotrigine and placebo groups.

Zoccali *et al*.[9] described a 24-week, randomized, double-blind, placebo-controlled study of lamotrigine (up to 200 mg/day) augmentation of a stable dose of clozapine (150-650 mg/day) in 60 patients with antipsychotic- and clozapine-resistant schizophrenia. In a completer (n=51) analysis, lamotrigine attenuated positive, negative, and general psychopathology symptoms as well as improved certain aspects of cognitive functioning without impacting on clozapine blood levels. One lamotrigine patient dropped out due to rash; otherwise, the medication was well tolerated.

Poyurovsky *et al*.[10] described an 8-week uncontrolled trial of lamotrigine (200 mg/day) augmentation in patients with schizophrenia (n=5) or schizoaffective disorder (n=6) and comorbid OCD. In the 9 patients who completed the study, Y-BOCS scores showed a small but statistically significant decline from a mean of 23 at baseline to a mean of 17 at treatment endpoint. Five patients (45%), all schizoaffective, showed at least 35% attenuation of Y-BOCS ratings. Schizophrenic symptomatology did not alter with lamotrigine.

These modestly encouraging studies notwithstanding, two large, well-conducted, randomized, double-blind, placebo-controlled trials (the gold standard in research) yielded negative outcomes. These trials were described by Goff *et al*.[11] Both studies were 12 weeks in duration and examined flexibly dosed lamotrigine (100-400 mg/day) augmentation in schizophrenic patients with stable, residual psychotic symptoms that had not responded to atypical antipsychotic medication. In the first study (n=217), negative symptoms and CGI-I ratings unexpectedly improved more with placebo than with lamotrigine. In the second study (n=212), a cognitive composite score improved more with lamotrigine than with placebo. In neither study did lamotrigine outperform placebo on positive symptom or PANSS total scores. Dropout rates were similar in lamotrigine and placebo groups.

Of modest concern are the reports that lamotrigine may rarely worsen psychosis. For example, Brandt *et al*.[12] reported that 6 out of about 1400 epileptic patients developed a psychotic disorder during treatment with lamotrigine. Chan *et al*.[13] also reported worsening of psychotic symptoms after lamotrigine addition in a patient with schizophrenia. Konstantakopoulos *et al*.[14] reported that 3 patients with paranoid schizophrenia experienced worsening of psychotic symptoms within 4-6 weeks of initiation of lamotrigine augmentation; the patients improved within 2 weeks of the withdrawal of lamotrigine. These stray reports could represent a coincidental rather than a cause-effect relationship.

Does lamotrigine alter antipsychotic levels? Bienentreu and Kronmuller[15] reported that lamotrigine increased risperidone levels in one patient; no mechanism for the interaction appeared evident. However, Spina *et al*.[16] found that lamotrigine (up to 200 mg/day) did not alter steady state blood levels of atypical antipsychotic medications or their metabolites in schizophrenic or bipolar patients receiving clozapine (200-500 mg/day), olanzapine (10-20 mg/day), or risperidone (3-6 mg/day); whereas an increase in olanzapine levels was statistically significant, the absolute magnitude of increase was too small to be clinically significant.

What is the take-home message? Many studies with sample sizes in the region of n=50 or less have described improvement in positive symptom and general psychopathology ratings with lamotrigine augmentation of antipsychotic medication in patients with refractory schizophrenic illness. However, two large (n>200, each), randomized, double-blind, placebo-controlled studies found no such benefits. Thus, the usefulness of lamotrigine augmentation in medication-refractory schizophrenia remains to be established. It is theoretically possible that a subgroup of patients may improve, and also that some vulnerable patients may worsen with lamotrigine augmentation. Whether lamotrigine reduces OCD symptoms in schizophrenic patients with comorbid OCD requires formal study. Finally, lamotrigine probably does not influence antipsychotic blood levels; at least, not to a clinically significantly extent.
